# A case of *Capnocytophaga canimorsus* endocarditis in a non-immunosuppressed host: the value of 16S PCR for diagnosis

**DOI:** 10.1099/acmi.0.000235

**Published:** 2021-05-04

**Authors:** Mark McNicol, Peter Yew, Gwyn Beattie, Laura Loughlin

**Affiliations:** ^1^​ Royal Victoria Hospital, Belfast, BT 12 6BA, UK; ^2^​ South Eastern Health and Social Care Trust, Belfast, UK; ^3^​ Belfast Health and Social Care Trust, Belfast, UK

**Keywords:** *Capnocytophaga*, 16s PCR, endocarditis

## Abstract

*
Capnocytophaga canimorsus* is a rare cause of endocarditis and is particularly unusual in non-immunosuppressed hosts. It is associated with animal bites, particularly those from dogs. This case describes a healthy 59-year-old woman, with no identifiable risk factors or dog bite history, who presented with fever of unknown origin. Echocardiography demonstrated an aortic valve mass and root abscess, in keeping with endocarditis, requiring urgent valve replacement surgery. Eight sets of blood cultures were drawn in total; after prolonged incubation, one set grew *C. canimorusus*. There was initial uncertainty over this being the causative organism, given the lack of immunosuppression or dog bite history, but 16S PCR of the valve identified the same organism, permitting targeted treatment. This case highlights the value of valve 16S PCR as a diagnostic tool in endocarditis.

## Background

*Capnocytophaga canimorsus* is an organism that has been associated with fulminant sepsis following dog bites. It has been identified as a rare cause of infective endocarditis and the literature to date suggests that this condition is usually associated with immunosuppressed hosts [1]. It is fastidious, which can make culture unreliable and untimely for prompt, targeted antibiotic treatment. Here we present a case of endocarditis in an immunocompetent patient with no past medical history or history of a dog bite.

## Case presentation

A 59-year-old woman attended her General Practitioner (GP) with a 3-week history of lethargy, myalgia, pyrexia and night sweats. She had no significant past medical history and was not on any regular medications. Her most recent travel had been to Spain a few months previously, and she lived at home with her husband and dog, who were in good health. Her GP performed some blood tests, shown in Table 1, which prompted urgent referral for admission to hospital due to the notably raised white cell count and CRP.

**Table 1.** Blood testing from GP

Test (reference range)	Result
Haemoglobin (120–150 g l^−1^)	120
Total white cell count (4–10 10^9^ l^−1^)	26.4
Neutrophil count (2–7 10^9^ l^−1^)	23.67
Platelets (150–400 10^9^ l^−1^)	214
C-reactive protein (0–5 mg l^−1^)	92
ESR (1–20 mm h^−1^)	59
Sodium (133–146 mmol l^−1^)	128
Potassium (3.5–5.3 mmol l^−1^)	4.1
Chloride (95–108 mmol l^−1^)	88
Urea (2.5–7.8 mmol l^−1^)	3.4
Creatinine (45–84 umol l^−1^)	61

In the Emergency Department the patient was pyrexic with a temperature of 39.4 °C. Otherwise, her observations were normal. She had no audible murmurs and the rest of her examination was unremarkable.

Chest X-ray was normal and ECG showed sinus rhythm with a rate of 76 beats per minute and first-degree AV block. One set of blood cultures and urine cultures were sent and she was started on IV piperacillin/tazobactam for pyrexia of unknown origin. She was tested for influenza by PCR of a nasopharyngeal swab, which was negative.

Transthoracic echocardiography (TTE), carried out on day 2 of admission, demonstrated a mass on the aortic valve, which was further characterized by trans-oesophageal echocardiography (TOE), identifying a 1.8×0.9 cm mass with thickening of the aortic root, suspicious for root abscess.

On day 2 of admission, when the TTE raised the suspicion of endocarditis, she had seven further sets of blood cultures sent over a period of 72 h and was changed to IV amoxicillin 2 g 4 hourly, and gentamicin 3 mg kg^−1^ OD, as empirical treatment for native valve infective endocarditis.

**Fig. 1.**
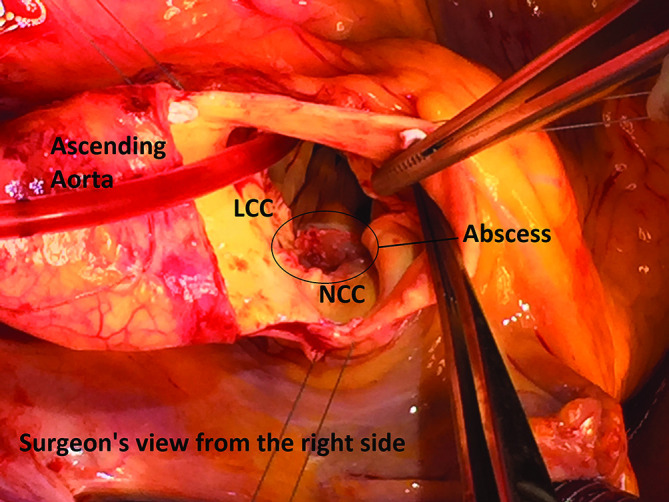
Intra-operative photograph of aortic valve taken from surgeons view. LCC, left coronary cusp. NCC, non coronary cusp. Photograph courtesy of Mr Gwyn Beattie, Consultant Cardiothoracic Surgeon, Royal Victoria Hospital, Belfast.

She was transferred to cardiac surgery on day 4 of admission and underwent aortic valve replacement surgery (Fig. 1). Procedure note from Cardiothoracics as follows: ‘Via oblique aortotomy, tri-leaflet valve was seen with a huge vegetation and perforation at the annulus. There was a small abscess cavity in the annulus at the non-coronary cusp. The native valve was excised, and the cavity was debrided easily, with rifampicin washout. A size 21 Inspiris Reslia bioprosthetic valve was implanted in annular position with 15× Ethibond 2/0 interrupted technique (5 Teflon pledgeted at the Non coronary cusp area). Aorta closed in single layer, using Haemoseal pledgeted sutures. De-aired. Cross-clamp off. Right Ventricular pacing wire inserted and checked. Weaned off Cardio-Pulmonary Bypass. Good valve function with no paravalvular leak and adequate de-airing confirmed on Trans-oesophageal ECHO. Mediastinal and pericardial drains inserted. Protamine given, haemostasis checked/effected.’

The valve was sent by the cardiac surgeons to the microbiology laboratory for culture, and sent away for 16s PCR testing. It was not sent to histopathology.

**Fig. 2.**
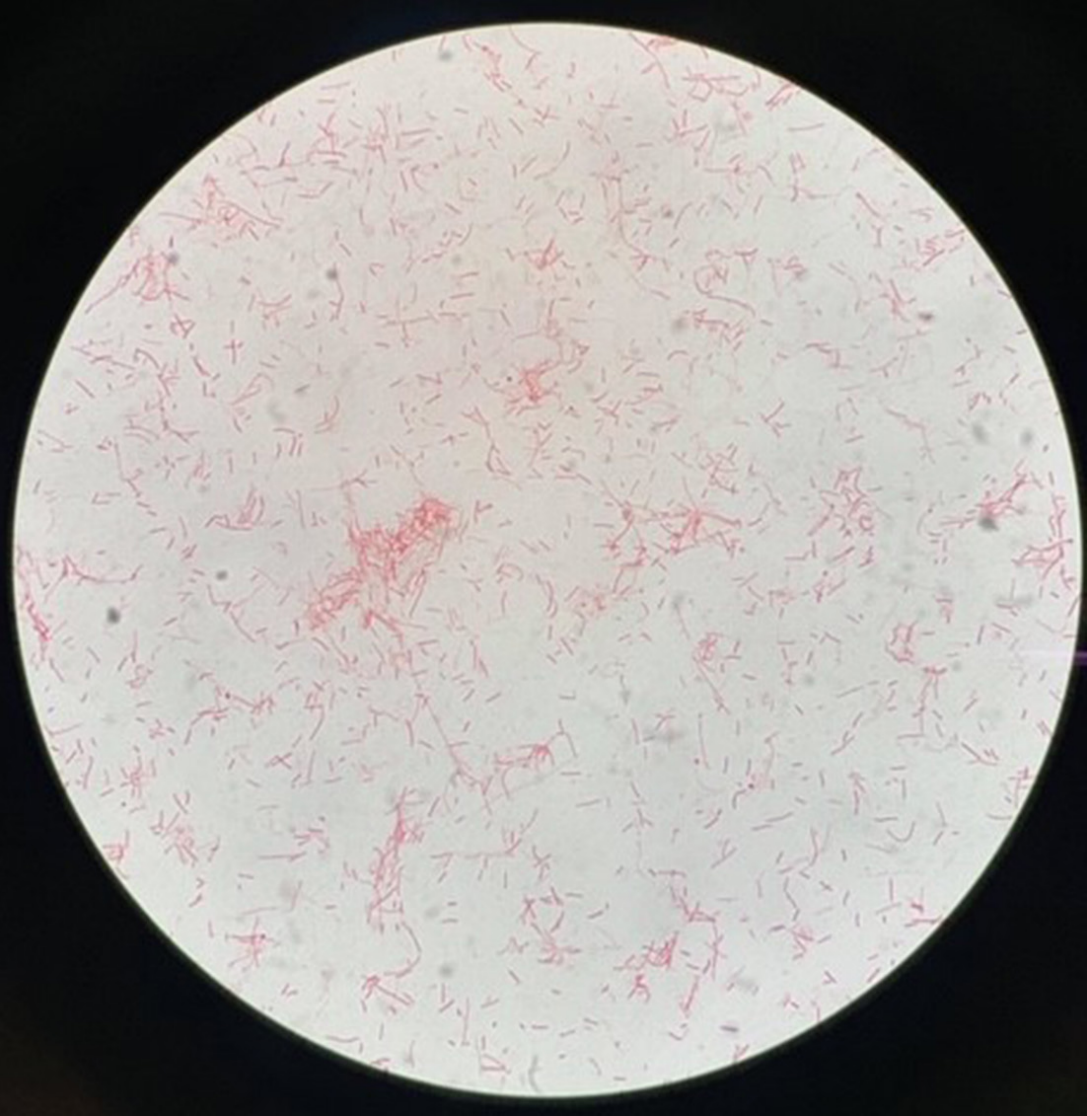
Gram stain of *C. canimorsus *. Courtesy of Dr Peter Yew, Consultant Medical Microbiologist, Ulster Hospital, Dundonald, Belfast.

On day 5 of admission, the aerobic bottle of the first set of blood cultures taken flagged positive after being incubated in Bact T/Alert 3-D (bioMérieux, France) for 3.5 days. Gram-negative rods were observed on Gram stain (Fig. 2). Given this information, the patient’s antibiotics were changed to ceftriaxone, vancomycin and gentamicin, whilst waiting for culture and identification of this organism. Subculture on Columbia blood agar yielded fine smooth, creamy white and non-haemolytic colonies. Species identification was performed using Vitek MS [matrix-assisted laser desorption/ionization time-of-flight mass spectrometry (MALDI-TOF MS)], which confirmed the organism as *C. carnimorsus*. As there were no recommended European Committee on Antimicrobial Susceptibility Testing (EUCAST) antibiotic breakpoints, E-test strips were set up and minimum inhibitory concentrations (MICs) were measured against EUCAST PK-PD (non-species-related) breakpoints. This organism was susceptible to gentamicin, ceftriaxone, penicillin, ciprofloxacin and meropenem. The remaining sets of blood cultures were all negative after 5 days of incubation.

Given the identification of this organism, the patient’s antibiotic therapy was changed to Meropenem, Vancomycin and Gentamicin pending susceptibility testing. The heart valve was cultured and was negative after 7 days of incubation in enrichment broth. The valve was also sent to the Public Health England reference laboratory for 16S rDNA real-time PCR identification. Report: this identified *Capnocytophaga* species*,* with data indicating a 99 % sequence homology to *C. cynodegmi, C. canimorsus* and *C. stomatis*, providing robust evidence to confirm the responsible pathogen. The patient’s treatment was, therefore, targeted towards *C. canimorusus,* based on the antibiotic susceptibility test results (Table 2) obtained through E-test, which were interpreted using EUCAST non-species-specific PK-PD breakpoints. Her antibiotic therapy was changed to IV benzylpenicillin, to complete 4 weeks of treatment.

**Table 2.** Minimum inhibitory concentrations (MICs) for antibiotics tested against *C. canimorsus* isolate

Antibiotic tested	MIC	Interpretation
Penicillin	0.047	S
Ceftriaxone	0.094	S
Gentamicin	12	S
Ciprofloxacin	0.032	S
Meropenem	0.003	S

The patient improved clinically and was discharged home after completion of treatment and a repeat ECHO prior to discharge showed normal left ventricular function and prosthetic aortic valve, with a repeat ECHO and follow-up planned in 1 year.

## Discussion

*C. canimorsus* is a slow-growing, fastidious Gram-negative bacillus that is part of normal oral flora in dogs. Patients at greatest risk of infection with *C. canimorsus* are those with anatomic or functional asplenia, heavy alcohol use, or hepatic cirrhosis. A review of 484 patients with laboratory-confirmed infection found that 87 % of patients had reported significant contact with dogs or cats, 60 % of which were bites [2].

In a 2019 case report of *C. canimorsus* endocarditis, the authors summarized all 18 previously published cases. In 12 of these the patient had animal contact with a dog, 1 had contact with a lion, and 5 had no disclosed animal contact [3]. On questioning, our patient owned a dog, but reported no bites. She had no underlying health problems, conditions predisposing to endocarditis or alcohol abuse. She had no clinical localizing signs, examination of her oral cavity by the dental team was unremarkable, and CT imaging of her abdomen showed no source of infection. There was diagnostic uncertainty when only one blood culture out of eight in total isolated the organism. We were reluctant to target *Capnocytophaga* alone as the causative organism of endocarditis, given the lack of host immunosuppression or dog bite history and the possibility of blood culture contamination, therefore antibiotic therapy was initially broad spectrum. 16S PCR of the heart valve provided the key supporting evidence for confirmed *C. canimorsus* endocarditis. This allowed focused, narrow-spectrum treatment with benzyl penicillin, which was an important stewardship measure. Additionally, this treatment regimen conferred reduced risk of adverse drug reactions. This case underlines the importance of 16S PCR investigation of the heart valve in endocarditis, particularly if blood cultures are negative or do not provide sufficient evidence to confirm the responsible pathogen. This is not a routinely available test in the majority of UK laboratories, often requiring referral to a reference laboratory, meaning that it is possibly underutilized in clinical practice. The case also underlines the importance of 16S PCR in other culture-negative infections on samples taken from clinical sites, such as CSF, which have been reported in the literature to diagnose *Capnocytophaga* species infections [4, 5].

As shown in Table 2, the organism was found to be susceptible to beta-lactam antibiotics and was likely to have been covered by the empirical regimen from the commencement of treatment. However, the time from presentation to time of availability of susceptibility test results was 15 days. This highlights the difficulty in establishing targeted treatment for this organism. The organism only grew in one blood culture, which was taken prior to antibiotic therapy. Cultures taken 48 h into antibiotic treatment were negative, which emphasizes the importance of obtaining multiple blood cultures without antibiotic exposure in the work-up for endocarditis. The authors postulate that the most likely source of infection was from the patient’s own dog, but accept that this cannot be proven.

In conclusion, *C. canimorsus* is an unusual and not often considered cause of endocarditis in a non-immunosuppressed host. This case highlights the use of 16s PCR on heart valves as an important diagnostic tool in endocarditis.
